# The comparative study of a homogeneous and a heterogeneous system with green synthesized iron nanoparticles for removal of Cr(VI)

**DOI:** 10.1038/s41598-020-64476-5

**Published:** 2020-04-30

**Authors:** Bo Guo, Meiling Li, Sai Li

**Affiliations:** College of Environmental Science and Engineering, Taiyuan University of Technology, Taiyuan, Shanxi 030024 P.R. China

**Keywords:** Environmental sciences, Chemistry, Materials science

## Abstract

Green iron nanoparticles (G-nZVI) were synthesized *in situ* by adding grape-seed extracts and Fe^2+^ solution simultaneously. The performances for the removal of Cr(VI) were compared in a homogeneous system by original G-nZVI (in suspension) with in a heterogeneous system by treated G-nZVI. The characterization of TEM, SEM, XRD, FTIR and XPS show that G-nZVI is the formation of Fe°-iron oxide core-shell nanoparticles with organic matters in the extracts as capping/stabilizing agents. The same excellent performances on the removal of Cr(VI) were observed in the both systems and the adsorption capacity was from 78.3 to 166.7 mg (Cr)·g^−1^ (Fe) with the increase of initial Fe^2+^ concentrations. The pseudo second-order model described the adsorption process excellently and both pseudo first-order and pseudo second-order models fit the reduction process well. It illustrated that the reaction included prompt adsorption and simultaneous redox process. Moreover, the results of thermodynamics study (ΔG° < 0, ΔH° > 0, ΔS° > 0) revealed that the adsorption was a spontaneous and endothermic process. It is obvious that the systhesis of original G-nZVI in the homogeneous system is more simple, rapid, cost-effective and suitable for *in situ* uses. It holds a great potential for remediation of soil and water.

## Introduction

Chromium is one of the most common heavy metals because of its chemical stability and low biodegradability^1–3^. Hexavalent chromium (Cr(VI)) is 100-fold times more toxic than trivalent chromium (Cr(III)) and highly soluble in water^4^. Hence, Cr(VI) can get into aquatic ecosystems such as underground water and contaminate drinking water^5,6^. Various chemical and physical treatments are employed for the Cr(VI) removal from the aqueous solution, such as membrane separation^7^, coagulation and flocculation^8^, adsorption and biosorption treatments^9–11^, Among the treatments, adsorption technique is advantageous due to its effectiveness, easiness in operating and less generation of chemical sludge.

In the current trend, nanotechnology has been widely preferred because of the huge specific surface area^12^. Nano-scale zero-valent iron (nZVI) plays a crucial role for environmental remediation due to its strong reducing power and its ability to adsorb many important contaminants such as heavy metals^13^. In fact, the most suitable method of Cr(VI) removal should include the reduction of Cr(VI) to Cr(III), since Cr(III) is significantly less toxic, much lower aqueous solubility and mobility than Cr(VI). Hence, nZVI based techniques have been widely employed in the removal of Cr(VI)^14,15^. However, nZVI particles tend to aggregate rapidly and thereby reduce the specific surface area and diminish particles reactivity. Various stabilization techniques have been applied to improve nZVI dispersibility, such as protective coatings^3,16^ and solid supports^9,10,17^. In recent years, green synthesis of nZVI has been received growing attention and developed as a promising alternative for chemical and physical methods, due to its advantages of a simple, rapid and cost-effective synthesis, biodegradable materials instead of toxic reagents (e.g. borohydride) and less agglomeration of nanoparticles^18–20^. In green synthesis of nZVI, a variety of materials from bio-renewable natural sources can be employed, which are even considered as wastes or do not have any added value in some cases^21–23^. In addition, the extracts of bio-materials with high water solubility, can also act as a nutrient source to improve complementary biodegradation^12,24,25^.

The green synthesis methods led to the concept that nZVI can be formed *in situ* by mixing the organic reductants and Fe^2+^/Fe^3+^ solution. Machado *et al*.^25^ demonstrated that green nZVI were synthesized *in situ* by adding the tree leaf extracts and Fe^3+^ solution simultaneously to ibuprofen-containing aqueous solutions, meanwhile, ibuprofen in the homogeneous system were degraded by the produced green nZVI. This simple and fast process is more economic than the traditional technology in the heterogeneous system, especially applied to the remediation of soils. However, in more cases, green nZVI particles should be separated from the solution (the mixture of Fe^2+^ / Fe^3+^ and extracts) and then dried to use in a heterogeneous system^26–28^.

The absence of studies concerning comparative properties and reactivity of green synthesized nZVI (G-nZVI) in the homogeneous and heterogeneous systems led to the present study. The objectives of this study were: 1) preparing two types of G-nZVI by grape-seed extracts to use in the homogeneous and heterogeneous systems respectively; 2) characterizing G-nZVI used in the two systems in terms of size, morphology, composition and structure; 3) evaluating and comparing reactivity of G-nZVI in the two systems based on the removal of Cr(VI); 4) investigating the corresponding kinetics and thermodynamics of Cr(VI) removal process and proposing the possible removal mechanism. The investigation would provide useful information for further development of G-nZVI.

## Materials and methods

### Reagents

All the chemicals including potassium dichromate, ferrous sulfate heptahydrate (FeSO_4_·7H_2_O) and ethanol were analytical reagent grade and used directly without further purification. These reagents were purchased from Tianjin Chemical Reagent Co. (Tianjin, China). Grapes were purchased from a local shop in Shanxi, China. Deionized (DI) water was used in all experiments.

### Synthesis of G-nZVI samples

Grape seeds were collected and washed with deionized water and then dried in an oven at 70 °C for 12 h. Dried grape seeds were grinded to obtain a powder with sizes below 1 mm. The extracts were prepared by heating 5 g powder in 100 mL mixture of ethanol and deionized water (1:1) at 70 °C for 90 min in a shaker bath (HZQ-X160, China), thereafter the extracts were vacuum-filtered and stored at 4 °C for further use. G-nZVI were synthesized by adding the grape–seed extracts and Fe^2+^ solution simultaneously. The immediate appearance of a black color indicated the reduction of Fe^2+^ ions. G-nZVI synthesized *in situ* were used in a homogeneous system. The prepared G-nZVI as above were separated and dried under vacuum at 50 °C for 12 h, and then kept in a nitrogen atmosphere prior to use. These G-nZVI would be used in a heterogeneous system.

### Characterization

G-nZVI in a dark colored solution were mounted on 300 mesh nickel grids and examined using a JEM 2010 Transmission Electronic Microscope (TEM; 120 kV) (Japan). Morphological characteristics were analyzed using a scanning electron microscopy (SEM) (JEOL/JSM-6700F). The phases of G-nZVI before and after reaction were characterized by powder X-ray diffraction (XRD) using an X-ray diffractometer (Rigaku D/max-2500, Japan) with monochromatized Cu Kα radiation, power setting of 40 kV, scan range from 5° to 90° at a scanning speed of 0.25°/min. FTIR spectra of the grape-seed extracts, G-nZVI before and after reaction were determined by a Fourier transform infrared spectroscope (FTIR Nicolet 5700, Thermo Corp., USA). Measurements were prepared by mixing 1% (w/w) specimen with 100 mg of KBr powder and pressed into a sheer slice.

### Batch Experiments

The reactivity of G-nZVI in the homogeneous and heterogeneous systems was evaluated respectively by assessing the extent of the reaction between G-nZVI and Cr(VI). The determination of Cr(VI) concentration was measured with the 1, 5-diphenylcarbazide method^29^. A fixed amount of G-nZVI was added into a series of 250 mL Erlenmeyer flasks with 100 mL Cr(VI) solution having an initial concentration of 25 mg·L^−1^. They were then kept at a given pH (in general pH 3) and temperature and agitated at 250 rpm. Certain flasks were withdrawn at fixed intervals until equilibrium was reached (90 min). Following reaction, the supernatant was filtered through 0.45 µm membranes to measure the residual concentration of Cr(VI).

## Results and Discussion

### TEM and SEM Characterization

**Figure 1:**
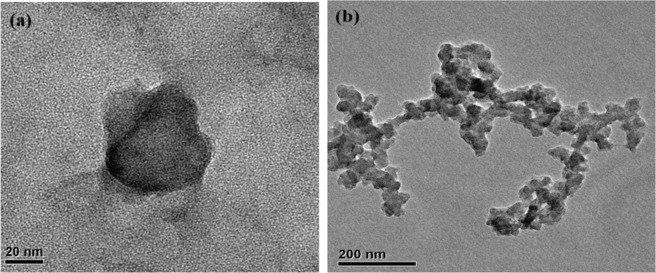
Representative TEM images of G-nZVI in a homogeneous system. (**a**) G-nZVI in suspension; (**b**) G-nZVI after reaction.

G-nZVI was produced *in situ* by mixing the grape-seed extracts with Fe^2+^ solution (0.10 M), and then added directly to a Cr(VI) solution. The morphologies and size of G-nZVI before and after reaction with Cr(VI) in the homogeneous system were determined by TEM as shown in Fig. 1. The representative TEM images showed that G-nZVI are quasi-spherical shaped nanoparticles with the sizes ranging from 30 to 80 nm, which is similar to the diameters of iron nanoparticles (from 20 to 80 nm) reported by Wei *et al*.^30^. The formation of nanoparticles is attributed to the existence of polyphenols or antioxidants in the extracts, such as flavonoids, alkaloids, chlorophylls^31^. These substances are responsible for the bioreduction of iron and stabilization of iron nanoparticles^30,31^. After reaction with Cr(VI) (Fig. 1(b)), chain-type agglomeration of nanoparticles was observed. It was probably due to the process of adsorption, and also redox reactions.

**Figure 2:**
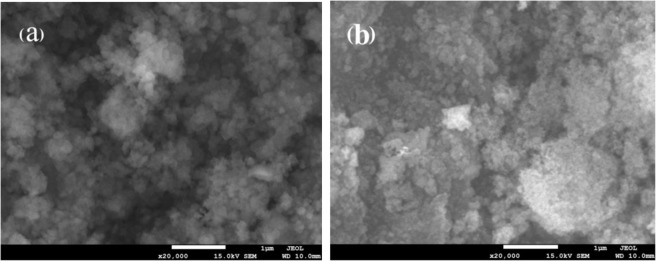
Representative SEM images of G-nZVI in a heterogeneous system. (**a**) separated G-nZVI and (**b**) G-nZVI after reaction.

G-nZVI in suspension (original ones, not treated) were separated and dried under vacuum at 50 °C for 12 h, and then added to a Cr(VI) solution. Figure 2(a,b) present examples of SEM images of G-nZVI before and after reaction with Cr(VI), respectively, in the heterogeneous system. Compared to G-nZVI in suspension, aggregations of G-nZVI can be seen in Fig. 2(a) and an increase in the dimensions of G-nZVI were observed. After reaction, larger agglomeration of particles can be seen in Fig. 2(b).

### XRD characterization

X-ray diffraction (XRD) was used to explore the crystalline structures of the as prepared materials. XRD patterns of the G-nZVI before and after the reaction with Cr(VI) in a heterogeneous system were investigated.

**Figure 3:**
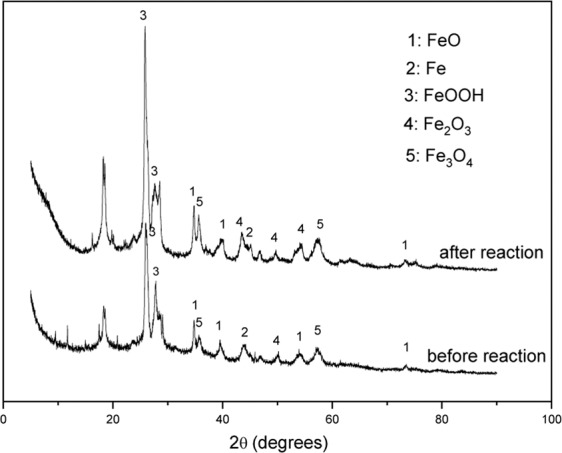
XRD patterns of G-nZVI.

As shown in Fig. 3, the diffraction peaks were identified to belong to FeO [PDF# 49-1447, Fe [PDF# 06-0696], FeOOH, α-Fe_2_O_3_ [PDF# 33-0664] and Fe_3_O_4_ [PDF# 26-1136]. The peak at 2*θ* = 17.96° was identified as the ingredient in polyphenols, which was confirmed in prior studies^32^. As seen in Fig. 3, characteristic peaks corresponding to FeO, Fe, FeOOH, Fe_2_O_3_ and Fe_3_O_4_ appear in patterns before and after reaction, indicating that G-nZVI contain iron oxide and iron oxyhydroxide. After reaction, the peak of Fe° weakens, meanwhile, characteristic peaks of iron oxide and iron oxyhydroxide strengthen slightly, demonstrating that chemical redox reaction occurred. Furthermore, the absence of chromium-containing molecules reflections indicates that chromium element is highly dispersed. Based on the above all, G-nZVI were probably oxidized to FeOOH or iron oxide after being exposed to air and water, and then formed a Fe°-FeOOH (and iron oxide)-polyphenols core-shell structure.

### FTIR characterization

**Figure 4:**
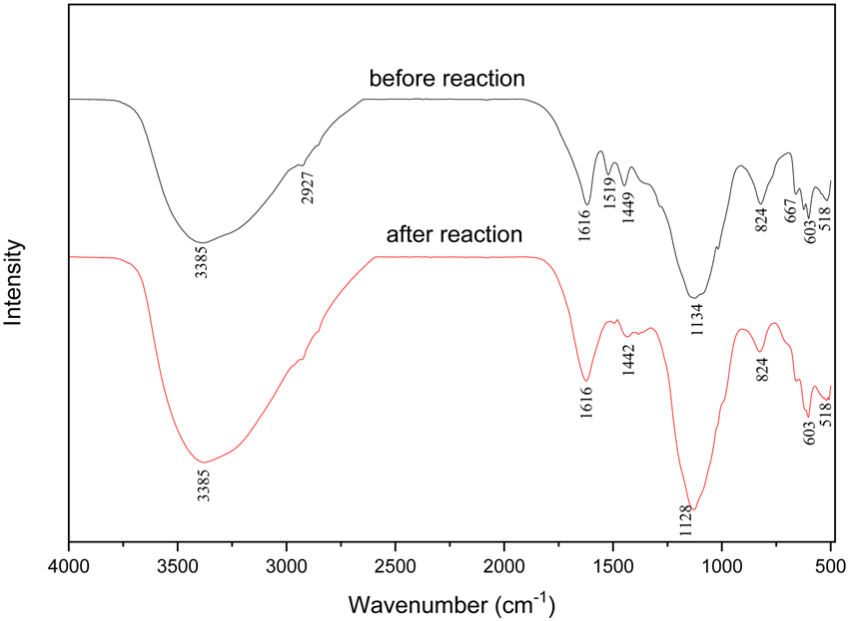
FTIR profiles of G-nZVI.

FTIR analysis was carried out to identify the interaction among biomolecules of grape-seed extracts and metal ions, responsible for formation and stabilization of iron nanoparticles. Figure 4 shows the spectra of G-nZVI before and after reaction in a heterogeneous system.

As shown in Fig. 4, strong bands at 3385 cm^−1^ and 1616 cm^−1^ are assigned respectively to O–H stretching vibrations and C═C aromatic ring stretching vibration^11,30,31^. The band at 1449 cm^−1^ before reaction and 1442 cm^−1^ after reaction corresponds to the in-plane bending vibrations of –OH in phenols. Peaks at 1134 cm^−1^ before reaction and 1128 cm^−1^ after reaction occurred due to vibrations in bond between C–O^33^. Thus, functional groups including phenols, carboxyl and carbonyl are confirmed on the surface of G-nZVI before and after reaction, which would sustain the stability of nanoparticles. Furthermore, adsorption bands at 824 cm^−1^ in the profile of G-nZVI before and after reaction, may be due to the partial deuteration of amine or carboxyl group^34^. While transmission peaks at 603 and 518 cm^−1^ presented in the profiles before and after reaction, could be assigned to Fe–O stretches of iron oxide^31^.

### XPS characterization

**Figure 5:**
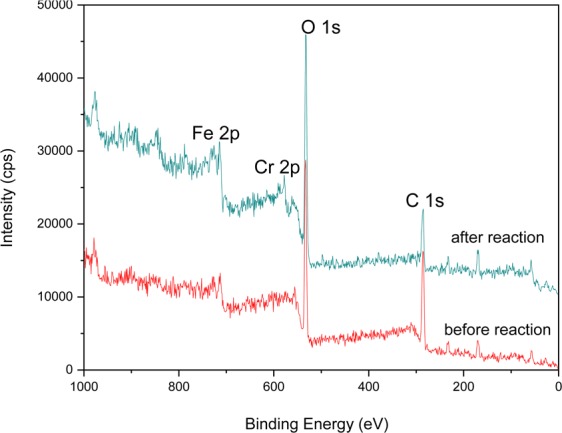
XPS patterns of G-nZVI.

XPS analysis was conducted to understand the surface chemical state of Cr, Fe, C and O in G-nZVI before and after the reaction with Cr(VI) in a heterogeneous system. Figure 5 presents the whole region scan of G-nZVI surface before and after reaction.

For fresh G-nZVI (before reaction), the principal elements at the surface are carbon (53.9 at%), oxygen (43.1 at%), and iron (3.0 at%). The C and O signals originated predominantly from the polyphenol groups and other C, O containing molecules in extracts^30,31^. After reaction with Cr(VI), new peaks around 580 eV emerge, which are designated to the photoelectron peaks of chromium. The main elements at the surface are carbon (40.5 at%), oxygen (51.3 at%), iron (4.8 at%) and chromium (3.3 at%). It indicates the uptake of chromium on the nZVI surface^35^.

**Figure 6:**
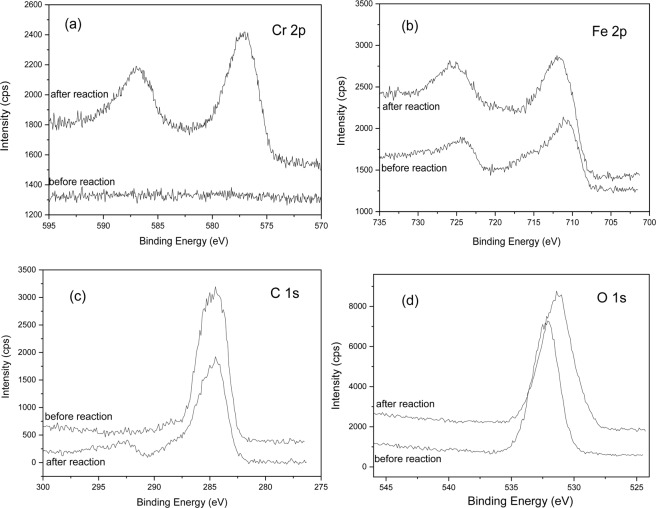
XPS survey of (**a**) Cr 2p, (**b**) Fe 2p, (**c**) C 1s and (**d**) O 1s for G-nZVI.

Detailed XPS survey on the regions of Cr 2p, Fe 2p, C 1 s and O 1s are presented respectively in Fig. 6(a–d).

As shown in Fig. 6(a), the photoelectron peaks for the chromium 2p3/2 and 2p1/2 centers at 577.0 and 586.8 eV after reaction, respectively, represent binding energies of Cr_2_O_3_ or Cr(OH)_3_^30,35^. The XPS results indicate that the reduction of Cr(VI) to Cr(III) is complete.

Fe 2p peaks around 711 and 725 eV in Fig. 6(b) correspond to the binding energies of 2p3/2 and 2p1/2 of ferric iron and ferrous iron. The main compound on the G-nZVI surface is iron ferrihydroxide (FeOOH)^30,35^. It is consistent with the results of XRD and FTIR. The absence of Fe° indicates oxidation of iron on the surface.

The photoelectron peak for C 1s at 284.5 eV in Fig. 6(c) corresponds to the polyphenol groups and C containing molecules in extracts. The decreasing peak height and peak area after reaction suggests the decrease contents of polyphenol and C containing molecules on the surface.

There is a broad O 1s peak around binding energy 532 eV in a curve of Fig. 6(d), which represents the binding energy of oxygen chemically or physically adsorbed water (≡OH_2_) on the surface of fresh G-nZVI. After the reaction with Cr(VI), O 1s peak emerges around 531 eV for OH as shown in curve b of Fig. 6(d)^35^. It indicates that the Cr(III) compound formed at the G-nZVI surface is Cr(OH)_3,_ not Cr_2_O_3_. As expected, the amount or the fraction of oxygen on the G-nZVI surface increased as a result of iron oxidation and hydration.

### Comparative study on removal of Cr(VI)

In the experiments, the performance of G-nZVI in the homogeneous and heterogeneous systems was evaluated respectively by removal efficiency of Cr(VI). The representative experiments were conducted in 250 mL flasks with 100 mL of a 25 mg·L^−1^ Cr(VI) solution. A 2 mL volume of G-nZVI in suspension or the corresponding amount of separated G-nZVI was added to the flask. Then the flask was capped to avoid dissolution of atmospheric oxygen that would decrease the nZVIs’ reactivity. The solution was continuously stirred for proper mixing. Samples were collected at selected time intervals and analyzed.

**Figure 7:**
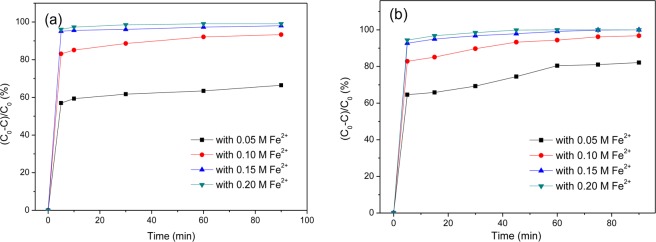
Removal efficiency of Cr(VI) using G-nZVI synthesized by different concentrations of Fe^2+^ solution. (**a**) in a homogeneous system; (**b**) in a heterogeneous system.

**Figure 8:**
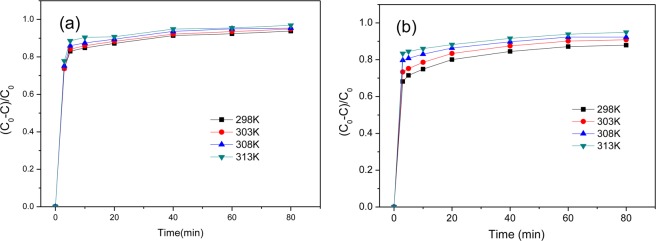
Removal efficiency of Cr(VI) by G-nZVI (with 0.1 M Fe^2+^) at different temperatures. (**a**) in a homogeneous system; (**b**) in a heterogeneous system.

As shown in Fig. 7(a,b), G-nZVI in the two systems are highly effective for removal of Cr(VI) in aqueous solution. Comparing the removal efficiencies of Cr(VI) in the two systems, no significant differences were observed for the samples synthesized with 0.10 M, 0.15 M and 0.20 M Fe^2+^ solution. While, G-nZVI synthesized with 0.05 M Fe^2+^ solution have a slightly higher efficiency in the heterogeneous systems than in the homogeneous ones. In all cases, reaction equilibrium was reached rapidly and G-nZVI in the homogeneous systems have better performances at the first 10 min, i. e. 83.1%, 95.1% and 96.1% of Cr(VI) was removed by G-nZVI with 0.10 M, 0.15 M and 0.20 M Fe^2+^, comparing 82.8%, 92.7% and 94.4% in the heterogeneous systems with corresponding Fe^2+^ solution, respectively, after the first 5 min reaction. The result shows that G-nZVI in a homogeneous system have the higher initial degradation rate, which is more favorable *in situ* remediation of water or soil. Furthermore, removal efficiencies of the samples increased with Fe^2+^ solution concentration in synthesis of iron nanoparticles, whereas barely increase was exhibited when a concentration of Fe^2+^ solution higher than 0.15 M was used. The effect of temperature on removal of Cr(VI) in the homogeneous and heterogeneous systems was investigated. As shown in Fig. 8(a,b), removal efficiencies of G-nZVI in the two systems increase with temperature rise from 298 to 313 K.

### Adsorption kinetics study

The results of characterization and batch experiments reveal that the removal of Cr(VI) may involve adsorption and redox reaction. In order to study the specific dynamics of the reaction process, two adsorption kinetics models were employed to fit the experimental data. One is the pseudo-first-order kinetics model, which described as follows^36^:

1 $${{\rm{In}}({\rm{q}}}_{{\rm{e}}}-{{\rm{q}}}_{{\rm{t}}}{)={\rm{Inq}}}_{{\rm{e}}}-{{\rm{k}}}_{1}{\rm{t}}$$

where *q*_*e*_ and *q*_*t*_ (mg·g^−1^) are the adsorption capacity of G-nZVI at the equilibrium time and at the different time t (min), respectively, and *k*_*1*_ (min^−1^) is the rate constant the pseudo first-order kinetics model for the adsorption.

Another one is the pseudo second-order kinetics model, described as bellow^17^:

2 $$\frac{{\rm{t}}}{{{\rm{q}}}_{{\rm{t}}}}=\frac{1}{{{\rm{k}}}_{2}{{\rm{q}}}_{{\rm{e}}}^{2}}+\frac{{\rm{t}}}{{{\rm{q}}}_{{\rm{e}}}}$$

where *k*_*2*_ (g·mg^−1^·min^−1^) represents the rate constant of the pseudo second-order kinetics model for the adsorption. Values of *k*_*2*_ and *q*_*e*_ can be calculated from the plot of *t/q*_*t*_ against *t*.

The fitting curves were shown in Figs. S1(a,b) and S2(a,b) respectively, and adsorption kinetics parameters were fitted and summarized in Table 1. The experimental data are much better fitted with the pseudo second-order model (r^2^ = 0.9987–0.9999) than the pseudo first-order model (r^2^ = 0.7448–0.9869). This indicates that a chemical adsorption is the dominated step to control the reaction rate in the homogeneous and heterogeneous systems due to the assumption of the pseudo second-order kinetics, which is based on the imagination that the adsorption rate is controlled by the chemical adsorption mechanism. The chemical adsorption process involves sharing and transferring of electrons between adsorbent and Cr(VI)^37^.

**Table 1:** Adsorption kinetics parameters for removal of Cr(VI) by G-nZVI in the homogeneous and heterogeneous systems. * *k*_*2*_ (mL·mg^−1^·min^−1^ for G-nZVI in a homogeneous system and g·mg^−1^·min^−1^ for G-nZVI in a heterogeneous system).

T(K)	Fe^2+^	Pseudo-first-order model		Pseudo-second-order model		
		k_1_ (min^−1^)	R^2^	k_2_ (mL·mg^−1^·min^−1^ or g·mg^−1^·min^−1^) *	R^2^	*q*_*e*_ (mg·g^−1^)
**In a homogeneous system**						
298K	0.05M	0.0535	0.7448	0.0072	0.9987	80.8
	0.10M	0.0577	0.9108	0.0113	0.9998	125
	0.15M	0.0509	0.8077	0.0253	0.9989	138.0
	0.20M	0.0335	0.8644	0.1013	0.9991	166.7
303K	0.10M	0.01099	0.9537	0.0133	0.9993	
308K	0.10M	0.01199	0.9576	0.0157	0.9992	
313K	0.10M	0.01234	0.9464	0.0169	0.9996	
**In a homogeneous system**						
298K	0.05M	0.0091	0.9452	0.2728	0.9959	78.3
	0.10M	0.0207	0.9869	0.4833	0.9994	111.7
	0.15M	0.0538	0.8495	1.0961	0.9999	123.7
	0.20M	0.0946	0.8595	2.1556	0.9999	166.7
303K	0.10M	0.01348	0.9271	0.7444	0.9996	
308K	0.10M	0.01416	0.9258	1.0114	0.9997	
313K	0.10M	0.01558	0.9883	1.0243	0.9996	

As the concentration of Fe^2+^ solution in the synthesis of G-nZVI increases from 0.05 to 0.20 M, *k*_*2*_ increases from 0.0072 to 0.1013 mL·mg^−1^·min^−1^ for G-nZVI in a homogeneous system and from 0.2728 to 2.1556 g·mg^−1^·min^−1^ for G-nZVI in a heterogeneous system, meanwhile, *q*_*e*_ also increases from 80.8 to 166.7 mg (Cr)·g^−1^ (Fe) for G-nZVI in a homogeneous system and from 78.3 to 166.7 mg·g^−1^ for G-nZVI in a heterogeneous system. The results suggest that both of the adsorption rate and the adsorption capacity increases with the rise of Fe^2+^ concentration in the two systems and G-nZVI in a homogeneous system has the slightly higher values of *k*_*2*_ and *q*_*e*_ than in a heterogeneous system. Furthermore, the adsorption capacity of Cr(VI) by G-nZVI is larger than by sugarcane bagasse (SMBC) (43.122 mg·g^−1^) reported by Yi *et al*.^30^.

With temperature rise from 298 to 313 K, *k*_*2*_ also increases from 0.0113 to 0.0169 mL·mg^−1^·min^−1^ for G-nZVI in a homogeneous system and from 0.4833 to 1.0243 g·mg^−1^·min^−1^ for G-nZVI in a heterogeneous system, indicating the endothermic process.

### Reaction kinetics study

Pseudo-first-order and the amended pseudo-second-order reaction models were employed to fit the experimental data. The pseudo-first-order reaction kinetics model, which was usually used to test the reduction process including nZVI-based nanoparticles^17^, could be described as the following equation:

3 $${\rm{In}}\frac{{\rm{c}}}{{{\rm{c}}}_{0}}=-\,{{\rm{k}}}_{{\rm{obs}}1}{\rm{t}}$$

where *c* is the concentration (mg·L^−1^) of Cr(VI) in solution, *k*_*obs*1_ (min^−1^) is the observed rate constant of the pseudo-first-order reaction that can be calculated from the slope of the line by plotting *ln(C/C*_*0*_*)* versus time.

The amended pseudo-second-order model can be described as follows^17^:

4 $${\rm{In}}\left(\frac{1}{{\rm{c}}}-\frac{1}{{{\rm{c}}}_{0}}\right)={{\rm{k}}}_{{\rm{obs}}2}{\rm{t}}$$

where *k*_*obs*2_ is the rate constant of the second-order reaction (L·mg^−1^·min^−1^). The value of *k*_*obs*2_ can be calculated from the slope of the line by plotting *ln(1/C-1/C*_*0*_*)* versus t (min^-1^).

The fitting curves and parameters were shown in Figs. S3(a,b) and S4(a,b) and Table 2. The correlation coefficient R^2^ were in the ranges of 0.9299-0.9981 and 0.9272-0.9899 for the pseudo first-order model, and 0.9402-0.9970 and 0.9026-0.9926 for the pseudo second-order model, in the homogeneous and heterogeneous systems respectively. Both pseudo first-order and pseudo second-order kinetics models can be used to fit the removal of Cr(VI) by G-nZVI. These results illustrated that the reaction included prompt adsorption and simultaneous redox process^17,37^. The rate constants, *k*_*obs*1_ and *k*_*obs*2_, increase from 0.0091 to 0.0968 min^−1^ and from 0.0121 to 0.0746 L·mg^−1^·min^−1^ for the homogeneous system, from 0.0026 to 0.0252 min^−1^ and from 0.0042 to 0.0257 L·mg^−1^·min^−1^ for the heterogeneous system, respectively, when the concentration of Fe^2+^ solution in the synthesis of G-nZVI increases from 0.05 to 0.20 M. This demonstrated that the removal rate depended on the concentration of Fe^2+^ and the active sites on the surface of G-nZVI^17^. The rate constant *k*_*obs*1_ and *k*_*obs*2_ slightly increased from 0.0133 to 0.0168 min^−1^ and from 0.0104 to 0.0156 L·mg^−1^·min^−1^ for the homogeneous system, from 0.0129 to 0.0145 min^−1^ and from 0.0107 to 0.0168 L·mg^−1^·min^−1^ for the heterogeneous system, respectively, when the temperature rose from 298 to 313 K. This indicates that the reaction of Cr(VI) with G-nZVI is an endothermic process.

**Table 2:** Reduction kinetics parameters for removal of Cr(VI) by G-nZVI in the homogeneous and heterogeneous systems.

T(K)	Fe^2+^	Pseudo-first-order model		Pseudo-second-order model	
		*k*_*obs1*_ (min^−1^)	R^2^	*K*_*obs2*_ (L·mg^−1^·min^−1^)	R^2^
**In a homogeneous system**					
298 K	0.05 M	0.0091	0.9449	0.0121	0.9443
	0.10 M	0.0133	0.9981	0.0129	0.9970
	0.15 M	0.0309	0.9707	0.0500	0.9732
	0.20 M	0.0968	0.9817	0.0746	0.9833
303 K	0.10 M	0.0150	0.9735	0.0134	0.9482
308 K	0.10 M	0.0157	0.9299	0.0144	0.9535
313 K	0.10 M	0.0168	0.9602	0.0145	0.9402
**In a heterogeneous system**					
298 K	0.05 M	0.0026	0.9560	0.0042	0.9487
	0.10 M	0.0104	0.9670	0.0107	0.9026
	0.15 M	0.0109	0.9899	0.0123	0.9926
	0.20 M	0.0252	0.9473	0.0257	0.9455
303 K	0.10 M	0.0135	0.9272	0.0152	0.9139
308 K	0.10 M	0.0142	0.9358	0.0158	0.9192
313 K	0.10 M	0.0156	0.9883	0.0168	0.9882

The activation energy can be calculated from Arrhenius equation, as shown below^38^:

5 $${{\rm{Ink}}}_{{\rm{obs}}1}=-\,\frac{{\rm{Ea}}}{{\rm{RT}}}+{{\rm{InA}}}_{0}$$

where *E*_*a*_ is the apparent activation energy (J·mol^−1^); *A*_*0*_ is the pre-exponential factor; *R* is the ideal gas constant (8.314 J·K^−1^·mol^−1^); and *T* is the reaction absolute temperature (K). According to Eq. (5), the values of *E*_*a*_ for the removal of Cr(VI) by G-nZVI are 11.6 and 18.9 kJ·mol^−1^ in the homogeneous and heterogeneous system respectively. This suggests that the removal of Cr(VI) by G-nZVI is a diffusion -controlled reaction^39^.

### Thermodynamics study

The experiments were performed at different temperatures to understand thermodynamics behavior of the removal of Cr(VI) by G-nZVI.

Thermodynamics parameters, change in standard Gibbs free energy (ΔG°), change in standard enthalpy (ΔH°), and change in standard entropy (ΔS°), could be calculated by the thermodynamics formulas as below^17,40^:

6 $${\Delta {\rm{G}}}^{0}=-\,{{\rm{RTInK}}}_{0}$$

7 $${{\rm{InK}}}_{0}=-\,\frac{{\Delta {\rm{G}}}^{0}}{{\rm{RT}}}=-\,\frac{{\Delta {\rm{H}}}^{0}}{{\rm{RT}}}+\frac{{\Delta {\rm{S}}}^{0}}{{\rm{R}}}$$

where, *K*_*0*_ is the equilibrium constant; ΔS° and ΔH° could be determined by plotting *lnK*_*0*_ versus *1/T*. The results of thermodynamics parameters had been listed in Table 3.

**Table 3:** Thermodynamics parameters for removal of Cr(VI) by G-nZVI in the homogeneous and heterogeneous systems.

Temperature(K)	LnK_0_	ΔG^0^ (KJ·mol^−1^)	ΔH^0^ (KJ·mol^−1^)	ΔS^0^ (KJ·mol−^1^)
**in a homogeneous system**				
298 K	3.722	−9.222	28.229	125.46
303 K	3.877	−9.766		
308 K	4.003	−10.251		
313 K	4.288	−11.159		
**in a heterogeneous system**				
298 K	3.408	−8.448	51.98	202.675
303 K	3.723	−8.589		199.8
308 K	3.916	−10.034		201.245
313 K	4.359	−11.35		202.235

As shown in Table 3, all the values of ΔG° are less than zero, revealing that the adsorption is spontaneous and Cr(VI) could be adsorbed from solution to the surface of G-nZVI. With the increase of temperature, the absolute values of ΔG° rise, indicating that the higher temperature is more favorable for the adsorption. In this study, the values of standard enthalpy ΔH° are greater than zero, illustrating that the adsorption of Cr(VI) by G-nZVI is an endothermic process. It is generally considered that ΔH^0^ is less than 20 kJ·mol^−1^ for absolute physical adsorption and in the range of 80–200 kJ·mol^−1^ for chemisorption. In the work, the values of ΔH^0^ are 28.229 and 51.980 kJ·mol^-1^ in the homogeneous and heterogeneous systems respectively, suggesting that the process is a combination of physical and chemical adsorption^17,41^. Moreover, all the values of standard entropy change ΔS^0^, are greater than zero, meaning that the removal of Cr(VI) by G-nZVI is an entropy increase process. Whereas, G-nZVI have larger values of ΔH^0^ and ΔS^0^ in the heterogeneous system than in the homogeneous system, which indicating the stronger interaction between Cr(VI) and G-nZVI in the heterogeneous system than in the homogeneous system.

### Possible removal mechanism

Based on above all the results, the possible mechanism of the removal Cr(VI) by G-nZVI (GnZVI) was proposed as below:

8 $${\rm{GnZVI}}+{\rm{Cr}}({\rm{VI}})\to {\rm{GnZVI}}-{\rm{Cr}}({\rm{VI}})$$

9 $${{\rm{Fe}}}^{0}+{\rm{Cr}}({\rm{VI}})\to {{\rm{Fe}}}^{2+}+{\rm{Cr}}({\rm{III}})$$

10 $${{\rm{Fe}}}^{2+}+{\rm{Cr}}({\rm{VI}})\to {{\rm{Fe}}}^{3+}+{\rm{Cr}}({\rm{III}})$$

First, Cr(VI) anions were adsorbed onto the surface of G-nZVI as shown in Eq. (8). Second, Cr(VI), which adsorbed in the active sites on the G-nZVI surface, took part in reducing reactions with Fe^2+^ or Fe^0^ as seen in Eqs. (9) and (10). Cr(VI) was reduced to Cr(III), meanwhile, Fe^0^ was firstly oxidized to Fe^2+^ and then Fe^2+^ continued the reaction as a reductant and was finally oxidized to Fe^3+^. With the change of pH, Cr(III) and Fe(III) could be transformed to Cr(OH)_3_ or Cr(III)/Fe(III) hydroxide^30^.

## Conclusions

In this study, excellent performances on the removal of Cr(VI) by either G-nZVI (synthesized *in situ*) in the homogeneous system or G-nZVI (separated) in the heterogeneous system, were proved in batch experiments. No significant differences were observed in the both systems. The characterizations of TEM, SEM, XRD, FTIR and XPS and the results of kinetics and thermodynamics study show that the Cr(VI) removal mechanism involved rapid adsorption and simultaneous redox. The systhesis of G-nZVI in the homogeneous system is more simple, rapid, cost-effective and suitable for *in situ* remediation. It holds great potential for remediation of soil and water.

## Supplementary information

Supplemental Material.
