# Supplementary material for: The comparative study of a homogeneous and a heterogeneous system with green synthesized iron nanoparticles for removal of Cr(VI)
Source: Sci Rep. 2020 Apr 30;10:7382. doi: 10.1038/s41598-020-64476-5 (PMC7193580; doi:10.1038/s41598-020-64476-5)
Supplement: Supplementary file 1 — Supplemental Material. [file 41598_2020_64476_MOESM1_ESM.docx]

**The comparative study of a homogeneous and a heterogeneous system with green synthesized iron nanoparticles for removal of Cr(VI)**

Bo Guo^^[[1]](#footnote-1)^*^, Meiling Li, Sai Li

*College of Environmental Science and Engineering, Taiyuan University of Technology, Taiyuan, Shanxi, 030024, P.R. China*

**Figure S1**. Pseudo-first-order adsorption kinetics for Cr (VI) removal by G-nZVI synthesized by different concentrations of Fe^2+^ solution. (a) in a homogeneous system; (b) in a heterogeneous system.

**Figure S2**. Pseudo-second-order adsorption kinetics for Cr (VI) removal by G-nZVI synthesized by different concentrations of Fe^2+^ solution. (a) in a homogeneous system; (b) in a heterogeneous system.

**Figure S3**. Pseudo-first-order reduction kinetics for Cr (VI) removal by G-nZVI synthesized by different concentrations of Fe^2+^ solution. (a) in a homogeneous system; (b) in a heterogeneous system.

**Figure S4**. Pseudo-second-order reduction kinetics for Cr (VI) removal by G-nZVI synthesized by different concentrations of Fe^2+^ solution. (a) in a homogeneous system; (b) in a heterogeneous system.

1. * Corresponding author: guobo@ tyut.edu.cn, Tel./Fax: +86-351-7238463 [↑](#footnote-ref-1)
